# Early Liver Metastasis from Early Gastric Adenocarcinoma with a Small Neuroendocrine Carcinoma Component: A Case Report

**DOI:** 10.70352/scrj.cr.25-0296

**Published:** 2025-09-23

**Authors:** Takahisa Hirano, Michitaka Honda, Soshi Hori, Hirohito Kakinuma, Ryuya Yamamoto, Kazuki Nishino, Masatoshi Noda, Hidetaka Kawamura, Nobuyasu Suzuki, Yoshinao Takano, Noriyuki Uesugi, Tamotsu Sugai

**Affiliations:** 1 Department of Surgery, Southern Tohoku General Hospital, Koriyama, Fukushima, Japan; 2 Department of Minimally Invasive Surgical and Medical Oncology, Fukushima Medical University, Koriyama, Fukushima, Japan; 3 Department of Pathology, Southern Tohoku General Hospital, Koriyama, Fukushima, Japan

**Keywords:** gastric neuroendocrine carcinoma, gastric adenocarcinoma, liver metastasis

## Abstract

**INTRODUCTION:**

Gastric neuroendocrine carcinoma (NEC) is a rare histological subtype of gastric malignancy. A small NEC component may coexist with gastric adenocarcinoma, making preoperative diagnosis challenging. Currently, no established treatment strategies exist for cases in which an NEC component is identified only in postoperative pathological findings.

**CASE PRESENTATION:**

An 82-year-old man underwent esophagogastroduodenoscopy during a routine health checkup and was diagnosed with early-stage gastric cancer. The lesion was a superficial tumor, 30 mm in diameter, located on the lesser curvature of the cardia. Biopsy revealed well-differentiated adenocarcinoma. The patient subsequently underwent laparoscopic proximal gastrectomy with D1+ lymph node dissection. Histopathological examination of the resected specimen revealed adenocarcinoma with an NEC component, accounting for approximately 10% of the tumor. The pathological stage was T1bN1M0 (Stage IB), and the patient did not receive adjuvant chemotherapy. Five months after the surgery, the patient developed anorexia and weight loss. CT revealed multiple liver tumors. Diagnostic laparoscopy with excisional biopsy of the liver lesion was performed, revealing no peritoneal dissemination. Histopathological examination confirmed that the liver tumors were metastatic NEC, indicating hepatic recurrence of gastric NEC. Given the patient’s rapidly deteriorating condition and a performance status of 3–4, aggressive chemotherapy was deemed inappropriate, and palliative care was initiated. The patient died of the primary disease 8 months after surgery.

**CONCLUSIONS:**

A small NEC component may be undetectable preoperatively. The prognosis of mixed tumors is dictated by the malignancy of the NEC component rather than its tumor burden. Therefore, clinicians should consider NEC-equivalent adjuvant chemotherapy and intensive surveillance.

## Abbreviations

CA 19-9
: carbohydrate antigen 19-9
CEA
: carcinoembryonic antigen
EGD
: esophagogastroduodenoscopy
ENETS
: European Neuroendocrine Tumor Society
EOB
: ethoxybenzyl
H&E
: hematoxylin and eosin
MiNEN
: mixed neuroendocrine non-neuroendocrine neoplasm
NANETS
: North American Neuroendocrine Tumor Society
N/C
: nuclear to cytoplasmic
NEC
: neuroendocrine carcinoma
UICC
: Union for International Cancer Control

## INTRODUCTION

Gastric NEC is a rare disease, accounting for only 0.1%–0.6% of all gastric cancers.^1,2)^ Histopathologically, NEC is characterized by a high frequency of lymphatic and venous invasion, reported in 73%–92.3% and 76.9%–81.5% of cases, respectively.^3,4)^ Clinically, it also tends to metastasize via lymphatic or hematogenous routes and is associated with a poor prognosis.^5–7)^ In some cases, NEC components coexist with gastric adenocarcinoma.^8)^ When NEC is not identified in preoperative endoscopic biopsy specimens, patients may undergo standard surgical treatment based on a diagnosis of adenocarcinoma. However, there are currently no established treatment guidelines for adjuvant therapy in cases where NEC is diagnosed only in postoperative pathological specimens. Herein, we report a rare case of early gastric cancer that was curatively resected based on a preoperative diagnosis of adenocarcinoma. Postoperative histopathological analysis revealed a minor NEC component, and the patient subsequently developed multiple liver metastases in the early postoperative period.

## CASE PRESENTATION

An 82-year-old man was referred to our hospital following an EGD performed during a routine health checkup, which revealed suspected gastric cancer. Physical examination findings were unremarkable. The patient was 165.6 cm tall and weighed 60.7 kg. Blood tests showed no leukocytosis or anemia, and the liver function was within normal limits. Tumor markers were also within the normal reference ranges: CEA, 2.7 ng/mL (normal <3.0); CA 19-9, 8.0 U/mL (normal <37).

**Fig. 1:**
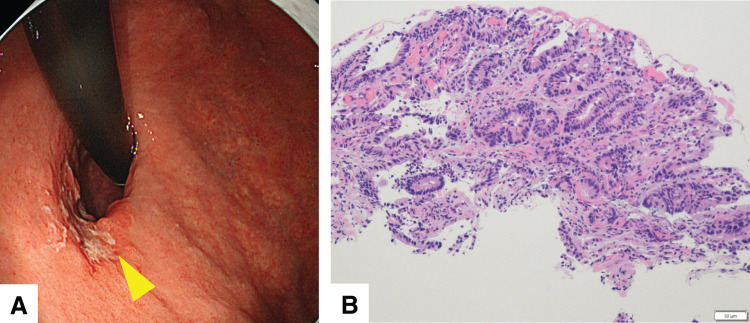
Preoperative EGD findings. (**A**) A superficially depressed and partially elevated (type 0–IIc+IIa) lesion located on the lesser curvature of the upper 3rd of the stomach. The arrowhead indicates the biopsy site. (**B**) H&E staining of the preoperative biopsy specimen showing a well-differentiated adenocarcinoma. Scale bar = 50 μm. EGD, esophagogastroduodenoscopy; H&E, hematoxylin and eosin

EGD revealed a superficial depressed and partially elevated lesion (type 0–IIc+IIa), measuring 30 mm in diameter, located on the lesser curvature of the upper 3rd of the stomach (**Fig. 1A**). Histopathological examination of a biopsy specimen revealed well-differentiated adenocarcinoma (**Fig. 1B**). Contrast-enhanced CT revealed no lymph node swelling or distant metastasis. Based on these findings, the patient was diagnosed with adenocarcinoma of the upper 3rd of the stomach, clinical stage T1bN0M0 (Stage I), according to the 8th edition of the UICC TNM classification. The patient underwent laparoscopic proximal gastrectomy with D1+ lymph node dissection.

**Fig. 2:**
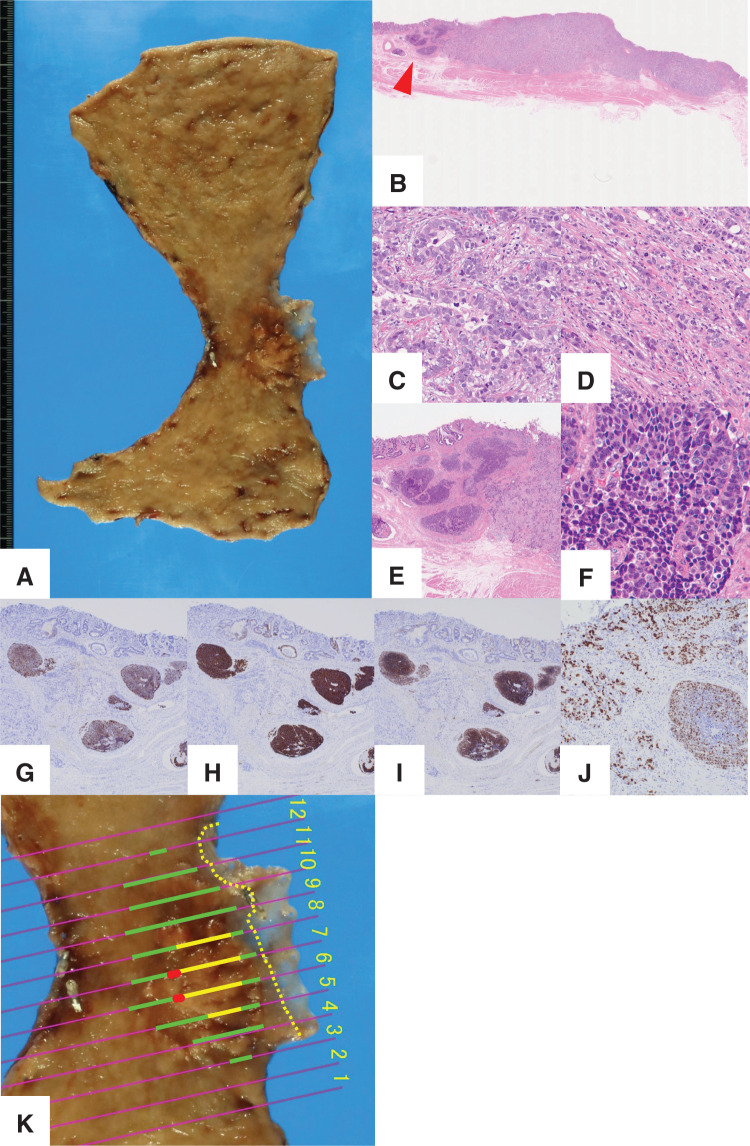
Pathological and immunohistochemical findings of the surgical specimen. (**A**) A superficial-type lesion, measuring 42 × 26 mm, located on the lesser curvature of the cardia. (**B**) H&E-stained macroscopic image showing predominant adenocarcinoma with submucosal invasion and a minor NEC component (arrowhead). (**C**) Moderately differentiated tubular adenocarcinoma (H&E, ×400). (**D**) Poorly differentiated adenocarcinoma, nonsolid type (H&E, ×400). (**E**) Atypical cells forming solid nests in the submucosa (H&E, ×40). (**F**) High N/C ratio (H&E, ×400). (**G**) Diffuse positivity for chromogranin A (×40). (**H**) Diffuse positivity for synaptophysin (×40). (**I**) Diffuse positivity for CD56 (×40). (**J**) Ki-67 labeling index was 58% (×100). (**K**) Green underline indicates the M cancer component, yellow indicates the SM component, and red indicates the NEC component. H&E, hematoxylin and eosin; M, mucosal; N/C, nuclear to cytoplasmic; NEC, neuroendocrine carcinoma; SM, submucosal

On gross examination, a 42 × 26-mm lesion was observed in the gastric cardia (**Fig. 2A**). Histopathological examination revealed that the majority of the tumor consisted of moderately differentiated tubular adenocarcinoma and poorly differentiated adenocarcinoma, nonsolid type, with invasion into the submucosa (**Fig. 2B**–**2D**). In part of the lesion, atypical cells with a high N/C ratio formed solid nests in the submucosa, exhibiting a sheet-like growth pattern. This component and the adenocarcinoma component were adjacent, with a clear demarcation and no transitional zone (**Fig. 2E** and **2F**). The cells of the solid nests were immunohistochemically positive for chromogranin A, synaptophysin, and CD56, with a Ki-67 labeling index of 58% (**Fig. 2G**–**2J**). Based on these findings, this component was diagnosed as NEC. The NEC component accounted for approximately 10% of the tumor (**Fig. 2K**). Furthermore, lymphatic and venous invasion were positive, and immunohistochemical staining confirmed that these invading cells were also NEC. The final pathological diagnosis was gastric adenocarcinoma with an NEC component, pT1bN1M0 (Stage IB).

On POD 7, the patient developed an ileus, which resolved with conservative treatment. The patient was discharged on POD 20. No adjuvant chemotherapy was given due to the patient’s advanced age and poor postoperative physical condition.

**Fig. 3:**
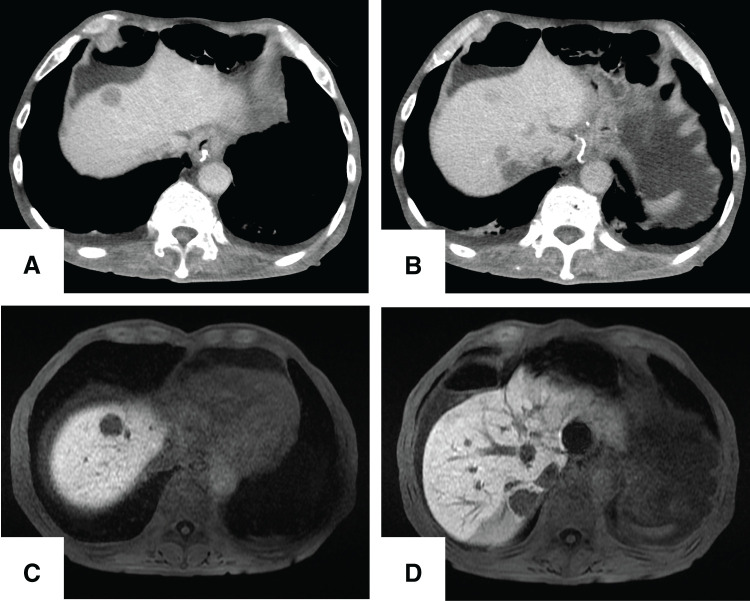
Imaging findings of multiple liver tumors. (**A**, **B**) Contrast-enhanced CT showing multiple hypodense liver lesions. (**C**, **D**) Ethoxybenzyl (EOB)-MRI (hepatobiliary phase) showing multiple hypointense liver lesions.

**Fig. 4:**
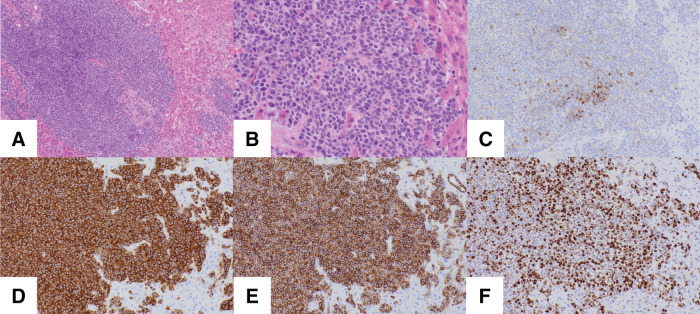
Pathological and immunohistochemical findings of the liver tumor. (**A**, **B**) H&E staining showing proliferation of cells with round nuclei ((**A**) ×100; (**B**) ×400). (**C**) Focal positivity for chromogranin A (×100). (**D**) Diffuse positivity for synaptophysin (×100). (**E**) Diffuse positivity for CD56 (×100). (**F**) Ki-67 labeling index was 60% (×100). H&E, hematoxylin and eosin

Five months after the surgery, the patient presented with anorexia and weight loss. Abdominal CT and MRI revealed multiple liver tumors (**Fig. 3**). A CT-guided biopsy of one liver lesion was attempted but yielded insufficient tissue for a conclusive analysis. Therefore, laparoscopic inspection and excisional biopsy of the liver tumor were performed. No peritoneal dissemination was noted. Histopathological examination revealed the proliferation of cells with round nuclei (**Fig. 4A** and **4B**). These cells stained positive for chromogranin A, synaptophysin, and CD56, with a Ki-67 labeling index of 60% (**Fig. 4C**–**4F**). The final diagnosis was metastatic NEC. These findings suggested that the liver metastases originated from the small-cell NEC component within the original gastric adenocarcinoma.

Owing to the patient’s rapid clinical deterioration and a performance status of 3–4, aggressive chemotherapy was deemed inappropriate. Palliative care was initiated, and the patient died of disease progression 8 months postoperatively.

## DISCUSSION

This case provides several important insights. First, gastric cancer often exhibits histological heterogeneity, and a small NEC component may be identified only after surgery. The presence of an NEC component can affect the prognosis, regardless of its volume. Second, there are no established postoperative management guidelines for postoperatively diagnosed NEC, and this remains an important area for future research.

The preoperative diagnosis rate of gastric NEC has been reported to be 18%–30%.^9,10)^ Several factors contribute to the difficulty in making a preoperative diagnosis of gastric NEC. First, its histogenesis is often attributed to the nodular proliferation of endocrine cells from the deep submucosal layer, which is difficult to capture with superficial endoscopic biopsies.^10,11)^ Second, NEC often appears similar to poorly differentiated adenocarcinoma on H&E staining. Our case was a typical example of this diagnostic challenge; the NEC component was localized to the submucosa at the periphery of the adenocarcinoma, and there were no macroscopic findings to suspect its presence during preoperative EGD. While techniques like boring biopsies and immunohistochemical staining are valuable when NEC is suspected,^12,13)^ their utility is limited when the lesion is endoscopically indistinguishable from a conventional adenocarcinoma, as it is impossible to determine where to target a deeper biopsy. This highlights an essential limitation in the preoperative diagnosis of such tumors.

Moreover, the NEC component formed solid nests with high cellularity, and the border with the adenocarcinoma was clear. These findings suggest that the NEC component represents an independent clone with distinct biological characteristics. The presence of vascular invasion and a high Ki-67 index indicates that the NEC clone had high-grade malignancy with significant proliferative and metastatic potential. In fact, the small NEC clone caused the multiple liver metastases that developed in the early postoperative period. These observations indicate that even a small NEC component can have a significant impact on prognosis if it is high-grade. Therefore, assessing the malignancy of a mixed tumor based solely on the proportion of its NEC component may be insufficient.

According to the 2022 World Health Organization classification,^14,15)^ a MiNEN of the digestive system is defined as a neoplasm in which the neuroendocrine and non-neuroendocrine components each constitute at least 30% of the tumor burden. However, this 30% cutoff has only been provisionally retained, and its clinical validity remains debatable.^15,16)^ Despite the NEC component accounting for only 10% of the tumor, thus not meeting the definition of the MiNEN classification, the patient’s postoperative course was rapidly fatal. Similarly, Furukawa et al.^17)^ described a case of gastric adenocarcinoma with a 10% NEC component that developed multiple liver metastases one year after curative resection. Park et al.^18)^ reported that a NEC component comprising ≥10% of a gastric adenocarcinoma was significantly associated with a poor prognosis. These findings indicate that the prognosis can be determined by even a small NEC component, revealing the limitations of the current quantitative definition of MiNEN.

There are no established guidelines for the management of gastric cancer with a small, postoperatively discovered NEC component. In contrast, for pure NEC, platinum-based chemotherapy is recommended as adjuvant chemotherapy.^19–22)^ In our case, no adjuvant chemotherapy was given due to the patient’s advanced age and poor postoperative physical condition. However, the aggressive clinical course of our patient suggests that adjuvant chemotherapy should be considered, even when the NEC component is small. Similarly, postoperative surveillance should also be reconsidered. According to the Japanese Gastric Cancer Treatment Guidelines,^23)^ the proposed follow-up model for Stage I gastric cancer includes imaging surveillance at 6 and 12 months postoperatively, and then annually. In contrast, the NANETS guidelines^20)^ and the ENETS guidelines^24)^ recommend imaging surveillance every 3–4 months for at least 2–3 years postoperatively for NEC. Therefore, in cases like ours, clinicians should adopt a follow-up protocol equivalent to that for NEC. Consequently, when a small NEC component is discovered postoperatively, short-term intensive surveillance based on the management of NEC may be warranted due to its potential prognostic impact.

## CONCLUSIONS

It is challenging to preoperatively diagnose a small NEC component in gastric adenocarcinoma. The clinical malignancy of such mixed tumors is dictated not by the quantitative proportion of the NEC component, but by its biological characteristics. Therefore, a thorough histopathological examination to identify any small NEC component is critical, and clinicians should adopt a postoperative management strategy for these patients, including both adjuvant chemotherapy and intensive surveillance, equivalent to that for NEC.

## DECLARATIONS

### Availability of data and materials

Data sharing is not applicable to this article as no datasets were generated or analyzed during the current study.
